# Comparison of the GPVI inhibitors losartan and honokiol

**DOI:** 10.1080/09537104.2019.1585526

**Published:** 2019-03-08

**Authors:** Marie-Blanche Onselaer, Magdolna Nagy, Chiara Pallini, Jeremy A Pike, Gina Perrella, Lourdes Garcia Quintanilla, Johannes A Eble, Natalie S. Poulter, Johan W.M. Heemskerk, Steve P Watson

**Affiliations:** 1 Institute of Cardiovascular Sciences, IBR Building, College of Medical and Dental Sciences, University of Birmingham, Birmingham, UK; 2 Department of Biochemistry, Cardiovascular Research Institute Maastricht (CARIM), Maastricht University, Maastricht, MD, The Netherlands; 3 Centre of Membrane Proteins and Receptors (COMPARE), Universities of Birmingham and Nottingham, The Midlands; 4 Institute of Physiological Chemistry and Pathobiochemistry, University of Münster, Münster, Germany

**Keywords:** CLEC-2, GPVI, GPVI, honokiol, losartan, platelets

## Abstract

Losartan and honokiol are small molecules which have been described to inhibit aggregation of platelets by collagen. Losartan has been proposed to block clustering of GPVI but not to affect binding of collagen. Honokiol has been reported to bind directly to GPVI but only at a concentration that is three orders of magnitude higher than that needed for inhibition of aggregation. The mechanism of action of both inhibitors is so far unclear. In the present study, we confirm the inhibitory effects of both agents on platelet aggregation by collagen and show that both also block the aggregation induced by the activation of CLEC-2 or the low affinity immune receptor FcγRIIa at similar concentrations. For GPVI and CLEC-2, this inhibition is associated with a reduction in protein tyrosine phosphorylation of multiple proteins including Syk. In contrast, on a collagen surface, spreading of platelets and clustering of GPVI (measured by single molecule localisation microscopy) was not altered by losartan or honokiol. Furthermore, in flow whole-blood, both inhibitors suppressed the formation of multi-layered platelet thrombi at arteriolar shear rates at concentrations that hardly affect collagen-induced platelet aggregation in platelet rich plasma. Together, these results demonstrate that losartan and honokiol have multiple effects on platelets which should be considered in the use of these compounds as anti-platelet agents.

## Introduction

Haemostasis is a finely regulated process triggered after vessel wall injury serving to curtail blood loss and to restore vascular integrity. One of the key components of haemostasis are platelets, which rapidly adhere and aggregate at sites of a lesion [1]. Glycoprotein VI (GPVI), a member of the immunoglobulin superfamily, is the major signalling receptor for collagen and fibrin [2–5]. GPVI is expressed on the surface membrane in complex with the FcRγ chain which contains an immunoreceptor tyrosine activation motif (ITAM) characterized by two YxxL/I sequences (where x is any amino acid) separated by 12 amino acids. Src kinases associated with the cytoplasmic tail of GPVI initiate phosphorylation of two conserved tyrosines in the ITAM of the FcRγ chain [6,7]. This leads to the recruitment and activation of Syk via its SH2 domains, and triggering of a phosphorylation complex consisting of adapter and signalling proteins that culminates in Ca^2+^ mobilisation and platelet activation [8,9].

The C-type lectin-like receptor, CLEC-2, has a single copy of a YxxL sequence known as a hemITAM. Phosphorylation of two CLEC-2 receptors leads to binding of Syk and initiation of a signalling cascade that is similar to that driven by the GPVI-FcRγ–chain [10–12]. To date, podoplanin is the only established endogenous ligand for CLEC-2. Podoplanin is expressed on the surface of a variety of cells including epithelial and stromal cells but is absent from vascular endothelial cells. Although its function in haemostasis is not clear, there is evidence that CLEC-2 play a pivotal role in arterial and venous thrombosis [13,14].

Losartan, an angiotensin II receptor antagonist, has been proposed to inhibit clustering but not binding of GPVI to collagen [15,16], leading to inhibition of platelet activation *in vitro* and reduced platelet accumulation after carotid injury in mice [17–20]. Honokiol is a natural bioactive molecule isolated from Magnolia species, which is used in traditional Chinese medicine. Honokiol is a multifunctional compound with many potential therapeutic properties, including antioxidant, anti-inflammatory, anti-cancer, anti-depressant and anti-neurodegeneration activities [21–23]. Honokiol also has anti-thrombotic effect, and has been shown to bind to GPVI at concentrations that are three orders of magnitude higher than those required for inhibition of platelet aggregation, suggesting an alternative mechanism of inhibition [24,25].In the present study, we have further interrogated the mechanism of action for both inhibitors.

## Material and Methods

### Reagents

Horm collagen and collagen diluent were purchased from Nycomed (Munich, Germany). CRP (ten glycine-proline-hydroxproline [GPO] repeats) was crosslinked as described [26]. Rhodocytin was purified in the Eble lab (University of Münster, Germany) from the crude venom of Calloselasma rhodostoma. The mouse monoclonal antibodies (mAbs) anti-phosphotyrosine clone 4G10 (05–321) and rabbit polyclonal anti-FcR γ-chain (06–727) were purchased from Merck Millipore (Watford, UK). The rabbit polyclonal antibody anti-Syk (sc-1077), the mouse mAbs anti-Syk 4D10 (sc-1240) and anti-FcR γ-chain (sc-390222) were purchased from Santa Cruz (Wembley, UK). All other reagents including losartan, honokiol and the anti-mouse IgG (Fc specific) F(ab′)_2_ fragment antibody were purchased from Sigma-Aldrich (Poole, UK), or came from described sources [3]. Losartan was dissolved in water and honokiol in DMSO. The mouse monoclonal mAb IV.3 against the low affinity immune receptor FcγRIIA was purified from the hybridoma obtained from the American Type Culture Collection. 1G5-Fab against Pan-GPVI was gift from Elizabeth Gardiner (Australian National University, Canberra, Australia).

### Platelet Isolation

Venous blood was taken from healthy volunteer using 3.8% (v/v) sodium citrate (1:9) as the anti-coagulant with informed consent according to the guidelines of the local ethics committee (ERN_11-0175). All steps of this study complied with the ethical principles according to the Declaration of Helsinki. Acid Citrate Dextrose (ACD, 1:10) was added to the blood. Platelet-rich plasma (PRP) was obtained by centrifugation at 200 *g* for 20 min at room temperature. Washed platelets were obtained by centrifugation at 1000 *g* for 10 min at room temperature using prostacyclin (2.8 μM) and resuspended in modified Tyrode’s-HEPES buffer (134 mMNaCl, 0.34 mM Na_2_HPO_4_, 2.9 mMKCl, 12 mM NaHCO_3_, 20 mM HEPES, 5 mM glucose, 1 mM MgCl_2_; pH7.3) Washed platelets were used at 2 × 10^7^/ml for static adhesion or 5 × 10^8^/ml for other studies.

### Platelet Aggregation

Washed platelets at 5 × 10^8^/ml were pre-treated for 5 min with different concentrations of losartan, honokiol or solvent controls prior to stimulation by collagen, rhodocytin, thrombin or mAb IV.3 crosslinked with F(ab’)_2_. Light transmission was recorded at 37°C with stirring (1200 rpm) in an aggregometer (Chrono-Log Stago, Havertown, Pennsylvania, USA). ATP secretion was monitored in washed platelets in parallel with platelet aggregation by adding firefly luciferase and luciferin (2 μM) and comparing the luminescence generated by platelet ATP release with an ATP standard.

### Platelet Spreading

Glass coverslips were coated in the presence of 10 μg/ml of collagen or fibrin generated as described previously [5]. Following washing with PBS, the coverslips were blocked with 5 mg/ml heat-inactivated bovine serum albumin (BSA) in PBS for 60 min. Washed platelets 2 × 10^7^/ml were incubated with honokiol (25 µM), losartan (25 µM) or solvent controls prior to be allowed to spread for 30 or 45 min, for human or mouse platelets respectively, at 37°C . The cells were then washed with PBS followed by fixation with paraformaldehyde (3.7%) for 10 min. For actin staining, the platelets were permeabilised with 0.1% Triton X-100 for 5 min and stained with Alexa-488-labelled phalloidin for 45 min in the dark. Platelets were imaged on a Zeiss Axiovert 200 M microscope. Fluorescence from platelets was analysed using ImageJ (NIH, Bethesda, USA). In each independent experiment, 5 random fields of view per condition were analysed, with at least 100 platelets in total per condition.

### Protein Phosphorylation Study

In presence of eptifibatide (9 µM), washed platelets 5 × 10^8^/ml were pre-treated for 5 min with honokiol (25 µM), losartan (25 µM) or solvent controls prior to activation by collagen (3 µg/ml), rhodocytin (150 µM) or thrombin (1 U/ml) with or without fibrinogen (200 µg/ml) at 37°C with stirring (1200 rpm) in an aggregometer for 60 seconds. Activation was terminated with 2x ice-cold lysis buffer (300 mM NaCl, 20 mM Tris, 2 mM EGTA, 2 mM EDTA, and 2% IGEPAL CA630 [NP-40 equivalent], pH 7.4, plus 2. mM Na_3_VO_4_, 100 mg/mL AEBSF [4-{2-aminoethyl} benzenesulfonyl fluoride hydrochloride], 5 mg/mL leupeptin, 5 mg/mL aprotinin, and 0.5 mg/mL pepstatin). Whole cell lysates (WCLs) were prepared by boiling a sample of lysate with sodium dodecyl sulfate (SDS) sample buffer. Syk was immunoprecipitated with 2 µg of Syk antibody (4D10) and incubated protein A-Sepharose beads overnight at 4°C. The beads were then washed, and proteins were eluted by boiling in SDS sample buffer. Immunoprecipitates (IPs) and WCLs were separated by SDS-polyacrylamide gel electrophoresis, electrotransferred, and western blotted. All primary antibodies were used diluted at 1/1000 in TBS-Tween-BSA 5% for overnight at 4°C. The secondary antibodies were used diluted at 1/10000 in TBS-Tween for 1 h at room temperature. Western blots were imaged with autoradiographic film.

### Cell/NFAT Assay

The following constructs and plasmids were used for GPVI transfections into cells for NFAT-luciferase assays as previously described [27]. Human GPVI sub-cloned into pcDNA3 with a C-terminal Myc tag and human FcRγ (untagged) in pEF6 were generated. The nuclear factor of activated T cells (NFAT)-luciferase reporter containing three copies of the distal NFAT site from the IL-2 promoter as described previously [28]. In brief, indicated amounts of DNA of each construct and 15 μg of NFAT-luciferase reporter construct were transfected by electroporation at 350 V and 500 µF microfarads into 2 × 10^7^ DT40 cells. The transfected cells were incubated with losartan at 25 µM or 250 µM and stimulated by collagen at 10 µg/ml for 6 h at 37°C. Luciferase activity was measured with a Centro LB960 microplate luminometer (Berthold Technologies, Germany).

### Platelet Spreading and Staining for STORM Imaging

For STORM imaging, 35 mm #1.5 (0.17 mm) glass bottomed MatTek dishes (MatTek Corporation, USA) were coated with 10 μg/ml Horm collagen diluted in manufacturer-supplied diluent or in 10 μg/ml cross-linked collagen related peptide (CRP-XL) using phosphate-buffered saline (PBS) and stored overnight at 4°C. Dishes were blocked with 5 mg/ml BSA for 1 h at room temperature then washed with PBS. Washed and rested human platelets, diluted to 2 × 10^7^/ml in modified Tyrode’s buffer were incubated with 2 μg/ml 1G5-Fab against Pan-GPVI for 5 min at 37°C. The labelled platelets were then incubated with 25 μM losartan or 25 μM honokiol or vehicle (PBS for losartan or DMSO for honokiol) for another 5 min at 37°C before being transfered onto the coated MatTek dishes. Platelets were allowed to spread for 45 min at 37 °C and rinsed once wit PBS to remove unbound platelets. Adhered platelets were fixed for 10 min with 10% neutral buffered formalin solution (Sigma, Poole, UK), followed by 5 min permeabilisation with 0.1% Triton X-100 in PBS. After PBS washes, the platelets were blocked for 1 hour at room temperature with 1% BSA + 2% goat serum (in PBS). 1G5-Fab-labelled platelets were secondary labelled with anti-mouse-Alexa647 antibody and stained for actin with Alexa488-phalloidin (both from ThermoFisherScientific; 1:300 diluition in block buffer) for 1 h at room temperature. Platelet samples were washed at least three times with PBS prior to imaging.

### STORM Imaging

Single-molecule images of platelet GPVI were acquired using a 100 × 1.49 N.A. TIRF objective lens on a Nikon N-STORM system in TIRF and dSTORM mode. The sytem was equipped with a Ti-E stand with Perfect Focus, Agilent Ultra High Power Dual Output Laser bed (containing a 170-mW 647-nm laser and a 20-mW 405 laser) and an Andor IXON Ultra 897 EMCCD camera. Fluorophore blinking was achieved by imaging the samples in a PBS based buffer containing 100 mM MEA-HCl, 50 µg/ml glucose oxidase and 1 µg/ml catalase as detailed in Metcalf *et al* [29]. Single colour (Alexa647) imaging was performed using the N-STORM emission cube with reactivation of fluorophore blinking achieved by increasing the 405 nm laser power by 5% every 30 sec. For each image, 20,000 frames were acquired using the Nikon NIS Elements v4.5 software with an exposure time of 9.2 ms, gain 300 and conversion gain 3. Image reconstruction to obtain localisation co-ordinates for each identified fluorescence blink was undertaken with the Nikon STORM analysis module 3.2 using drift correction and Gaussian rendering. For each condition, 5 different fields of view (FOV) from 3 independent experiments were captured. Only data points with a photon count>500 were included in the cluster analysis.

For the analysis of dSTORM data, localised data points within each FOV were grouped into clusters using density-based clustering of applications with noise (DBSCAN). The clustering was executed using the open-source software KNIME and the R package ‘dbscan’ (workflow available on request) [30]. The radius of local neighbourhood was set to 40 nm and 50 nm, and the minimum number of directly reachable points was set to 15 and 10, for CRP and collagen respectively. Cluster area was calculated by placing a circle of radius 30 nm over every detection in a cluster and calculating the union of these circles. This was estimated using a grid with pixel size 5 nm and image based dilation. The analysis was performed on whole FOVs and the quantitative cluster data was outputted as a spreadsheet. Graphs and statistics of the clustering data were calculated in Graphpad Prism 7.

### Whole Blood Thrombus Formation

Whole blood thrombus formation was assessed under flow conditions as described elsewhere [31]. In brief, glass coverslips were coated with 2 µl of 50 µg/ml collagen type I and mounted onto a transparent parallel-plate flow chamber. Blood samples treated with honokiol or losartan or vehicle were re-calcified with 3.75 mM MgCl_2_ and 7.5 mM CaCl_2_ in the presence of 40 µM active-site thrombin inhibitor PPACK, prior to experimentation. Recalcified blood samples were perfused for 6 min at wall shear rate of 1000 s^−1^ over the microspot surface. Representative phase-contrast microscopic images were captured using a fast line-scanning Zeiss LSM7 microscope equipped with 63x oil-immersion objective. Images were analysed using Fiji software [32]. Four outcome parameters were assessed: platelet surface area coverage, thrombus morphological score (0–5), thrombus contraction score (0–3) and platelet multilayer score (0–3) as previously explained [31,33]. Scoring was done by visual inspection of brightfield images based on a pre-defined score system. Morphological score indicates the aggregate formation by using a 5-point scale: 0: no platelet adhesion; 1: single platelet (>15) adhesion, 2: platelet monolayer; 3: small aggregates; 4: medium size aggregates; 5: large aggregates respectively. Thrombus contractility is appreciated based on a 3-point scale, where 0: no aggregate formation, 1: loose aggregates; 2: aggregates started to contract; 3: tightly packed, dense aggregates respectively. Multilayer score refers to thrombus volume on a 3-point scale, where 0: monolayer of platelets; 1: 2 layers of platelets; 2: multiple layer of platelets; 3: large, really high thrombi respectively.

### Data Analysis

Statistical analysis was realised by ANOVA with Turkey post-test. A P value of <0.05 was considered to be significant.

## Results

### Comparison of Losartan and Honokiol in Platelet Aggregation

**Figure 1.**
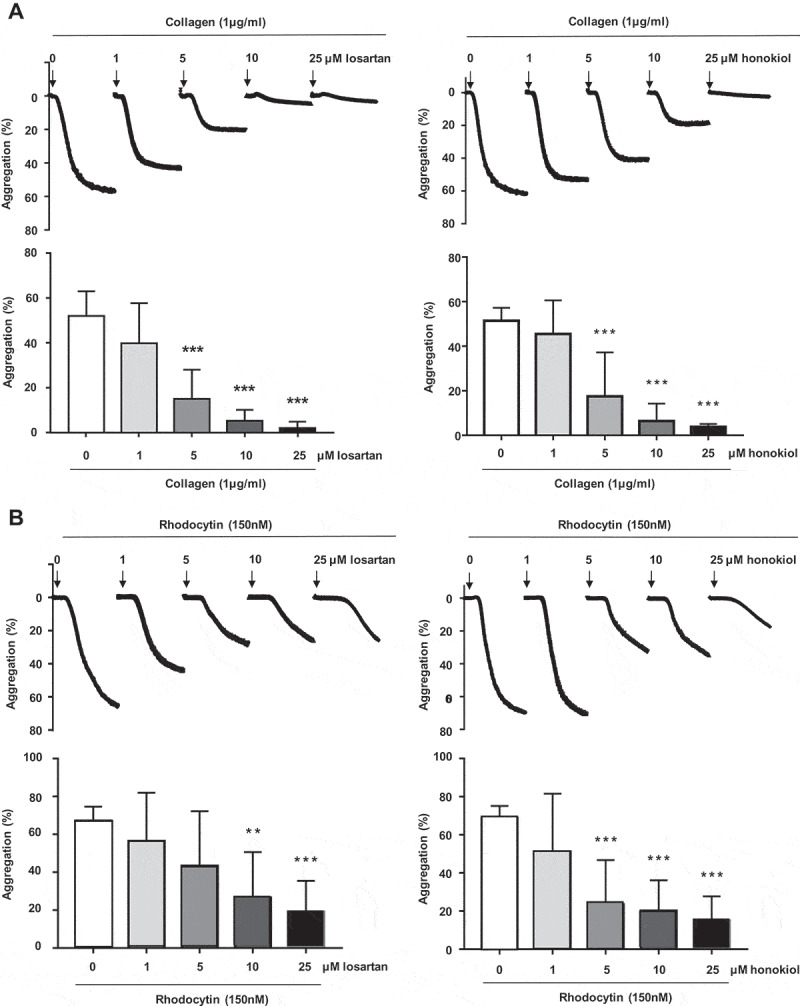
Losartan and honokiol dose-dependently reduce aggregation of washed platelets induced by collagen or rhodocytin. Washed platelets were pretreated with different concentrations of losartan or honokiol for 3 min before stimulation with a. collagen 1µg/ml or b. rhodocytin 150 nM. Bar graphs represent results from a total of 5 independent experiments and results are shown as mean ± SD **P < .01, ***P < .001.

**Figure 2.**
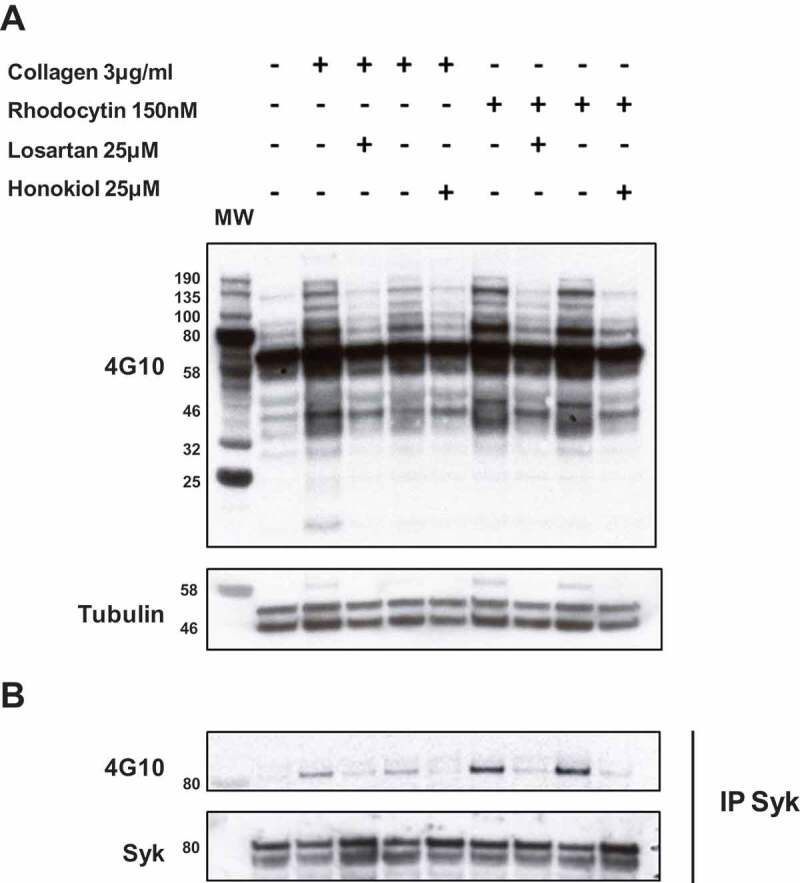
Losartan and honokiol inhibit platelet protein tyrosine phosphorylation, including Syk, induced by collagen or rhodocytin. Washed platelets were pre-treated with 25 µM of losartan or honokiol for 3 min before stimulation with collagen 3 µg/ml or rhodocytin 150 nM. Stimulations were stopped with addition of lysis buffer. a. whole-cell lysate (WCL) and b. immunoprecipitate (IP) of Syk. WCL and IP were separated by SDS-polyacrylamide gel electrophoresis and Western blotted for pTyr and Syk, respectively. The first lane shows the molecular weight markers (MW).The results are representative of 3 independent experiments.

In a previous study, platelet aggregation induced by 10 µg/ml of collagen was found to be inhibited by losartan with an IC50 of 6.3 µM [21] and by honokiol with an IC50 of 0.6 µM [26]. In Figure 1, we demonstrate, by comparing the two compounds, that losartan and honokiol dose-dependently inhibited aggregation of washed platelets induced by 1 µg/ml collagen with an IC50 of 3.7 µM for losartan and of 4.6 µM for honokiol. At these concentrations, neither inhibitor had an effect on platelet activation by thrombin or the thromboxane mimetic U46619 (data not shown). On the other hand, both losartan and honokiol delayed and reduced aggregation to the CLEC-2 agonist rhodocytin with IC50 values of 3.5 µM and 2.1 µM, respectively (Figure 1) and furthermore inhibited the platelet response to the low affinity immune receptor FcγRIIA over the same concentration range (Supplemental Figure 1). Collagen and rhodocytin induce a similar pattern of increase in tyrosine phosphorylation, which was abrogated by losartan or honokiol at approximately 10x their IC50 values (Figure 2). In addition, both inhibitors blocked tyrosine phosphorylation of the tyrosine kinase Syk (Figure 2).

**Figure 3.**
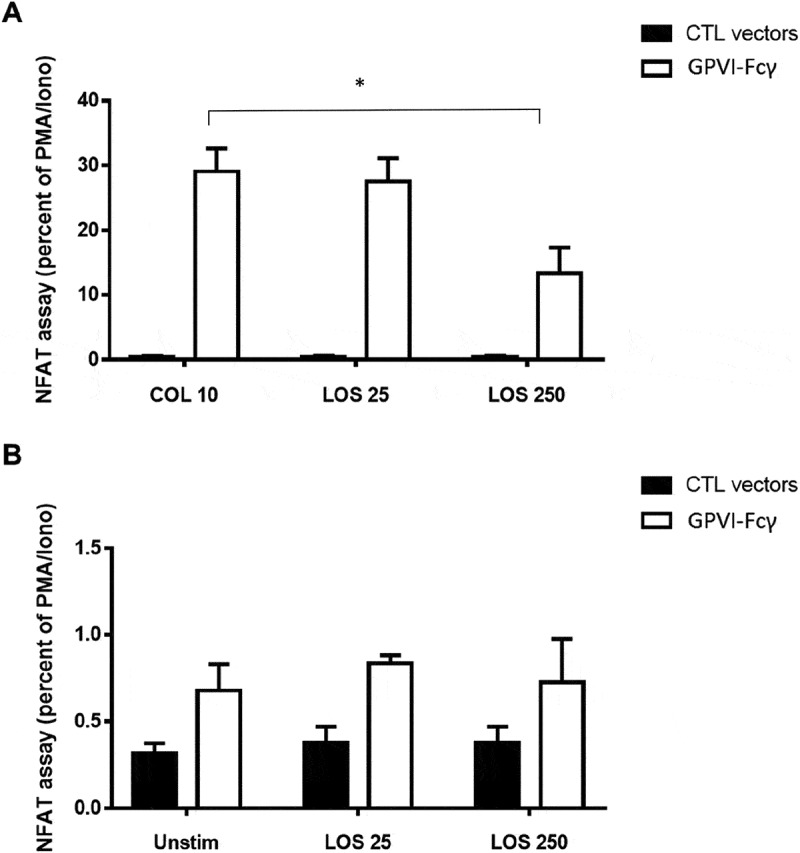
Losartan reduces signalling induced by collagen in transfected DT40 cells. DT40 B-cells were transfected with a NFAT-luciferase reporter construct, a β-galactosidase construct to control for transfection efficiency, and two GPVI and FcRγ-chain(Fcγ) expression constructs or empty control (CTL) vectors. Sixteen hours post-transfection, expression of GPVI was confirmed by flow cytometry (data not shown). The transfected cells were pre-treated with losartan (LOS) at 25 μM or 250 μM. They were either a. stimulated with collagen (10 μg/mL) or PMA (50 ng/mL) plus ionomycin (1 μM) or b. unstimulated. Six hours later, cells were lysed and assayed for luciferase and β-galactosidase. Luciferase data were normalized for β-galactosidase expression. Error bars represent the SEM from three independent experiments. * P < .001

Losartan and Honokiol were less potent in the presence of plasma, due to protein binding, requiring a concentration of 250 µM to achieve inhibition of aggregation (Supplemental Figure 2) [32]. At this concentration, losartan inhibited NFAT activation by GPVI and CLEC-2 in a transfected cell line model in the presence of serum (Figure 3a), but did not have an effect on constitutive signalling (i.e. ligand independentl signalling) of either receptor (Figure 3b). Honokiol could not be tested in the cell line model due to the effects of vehicle DMSO on NFAT activity.

### Losartan and Honokiol Do Not Block Platelet Spreading or Ligand Binding to GPVI

**Figure 4.**
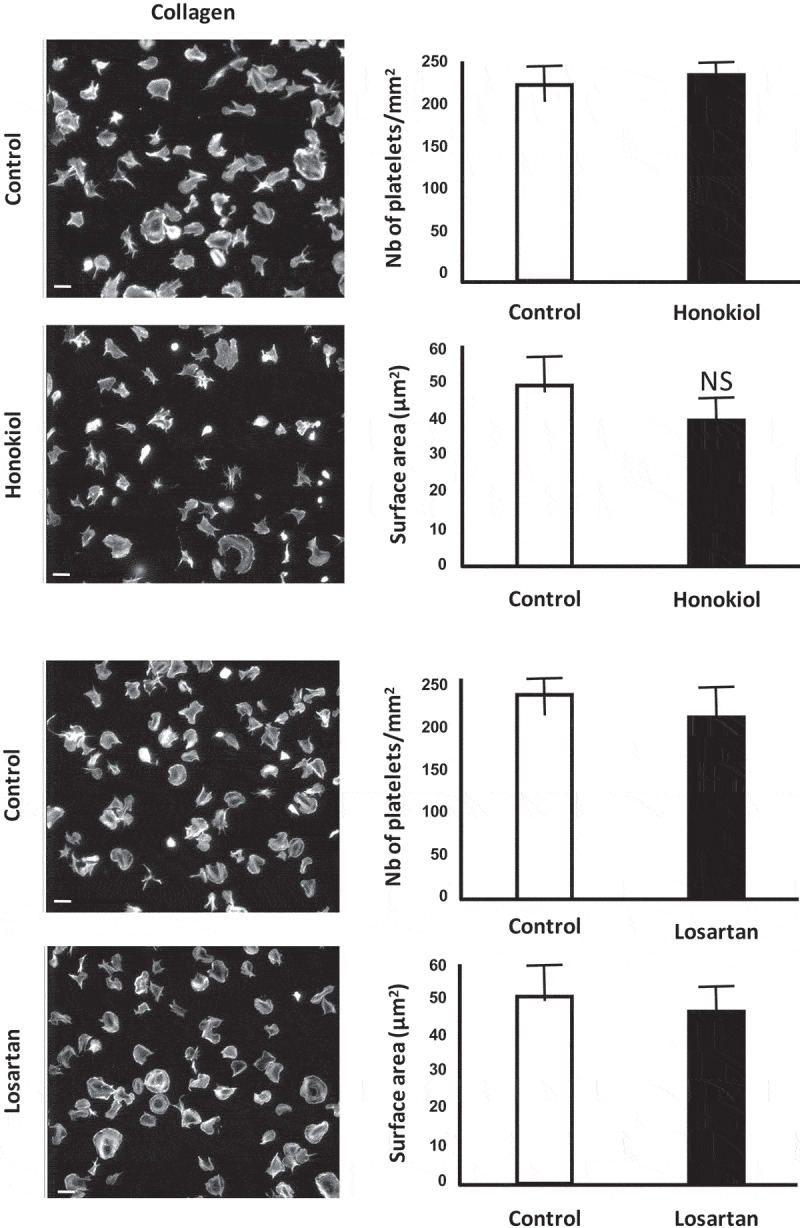
Losartan and honokiol do not affect platelet spreading on collagen. Glass coverslips were coated with collagen.Washed platelets were pre-treated with 25 µM of a. losartan or b. honokiol before spreading on collagen, followed by fixation and staining for actin with Alexa-488 phalloidin. Scale bar, 5 μm. Bar graphs illustrate quantification of the surface area covered with platelets per field, and the numbers of platelets counted per field. Dataare shown as mean ± SD and are representative of three experiments.

**Figure 5.**
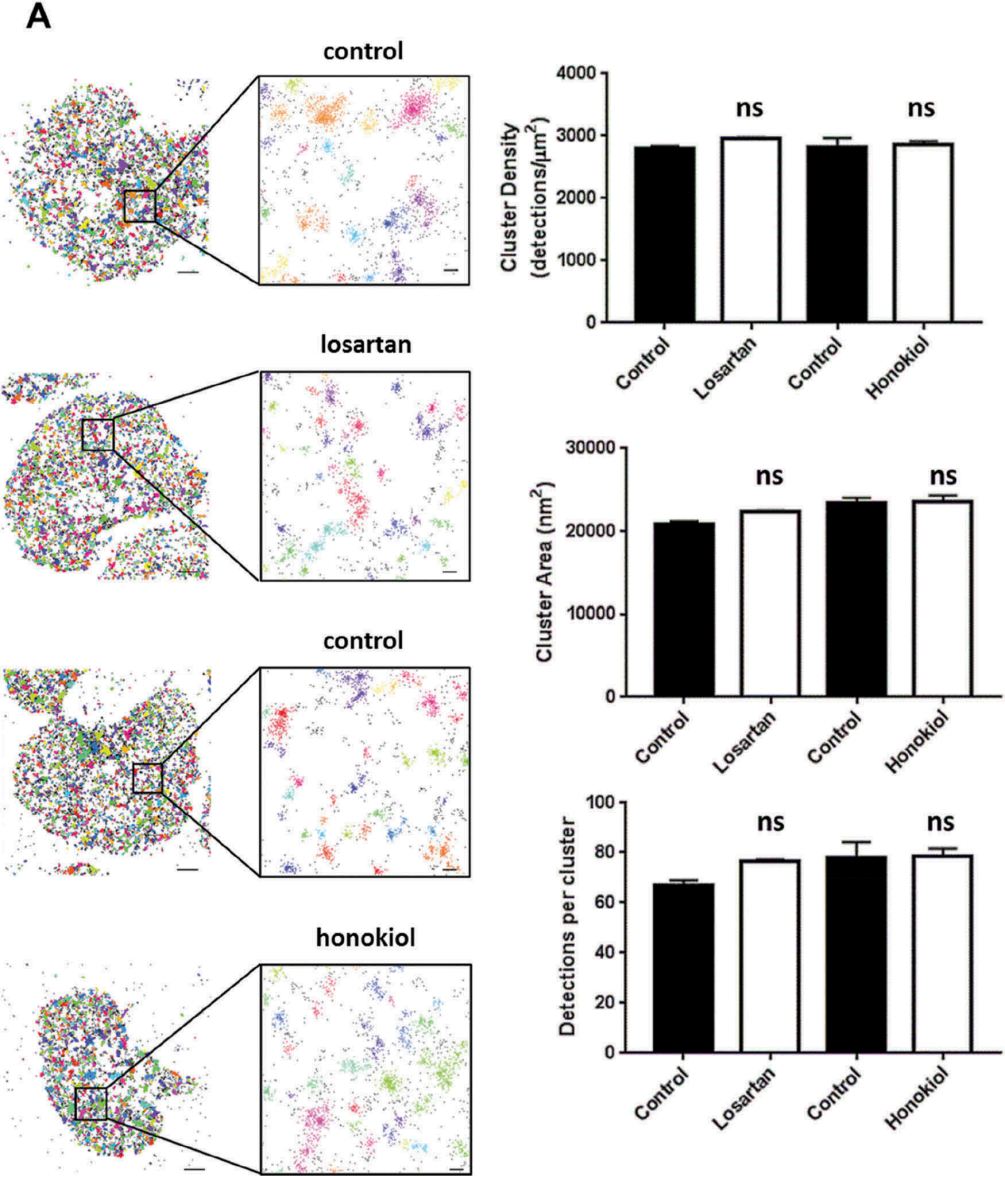
Losartan and honokiol do not affect clustering of GPVI. Glass coverslips were coated with CRP. Washed platelets were pre-incubated with 1G5 Fab (pan-GPVI), then pre-treated with 25 µM losartan, 25 µM honokiol or vehicle before spreading on CRP. Following fixation, GPVI was secondary labelled with mouse-Alexa647 and actin was labelled with Alexa488-phalloidin. The single molecule localisation data acquired by STORM was analysed for clustering using DBSCAN. Different colours represent different clusters (with black dots representing noise).For each condition, 5 different fields of view (FOV) from 3 independent experiments were captured. Representative cropped scatter plots for each condition show the output of the DBSCAN clustering algorithm (left panel). Scale bars: 1 µm and 0.1 µm. Bar graphs show quantification of cluster density, cluster area and number of detections per cluster. Mean ± SEM (n = 3) (right panel).

Losartan and honokiol (both used at 25 µM in Tyrode’s buffer) did not have significant effects on the adhesion or spreading of platelets on collagen (Figure 4). Similar results were observed at 100 µM (data not shown). Further, neither compound altered clustering of GPVI by CRP (Figure 5) or by collagen (not shown) as measured by single molecule localisation microscopy (dSTORM). This result is consistent with the lack of interference of either compound with GPVI-collagen binding, in an ELISA competitive binding assay using recombinant GPVI (Supplemental Figure 3) [21].

### Losartan and Honokiol Inhibit Thrombus Formation

**Figure 6.**
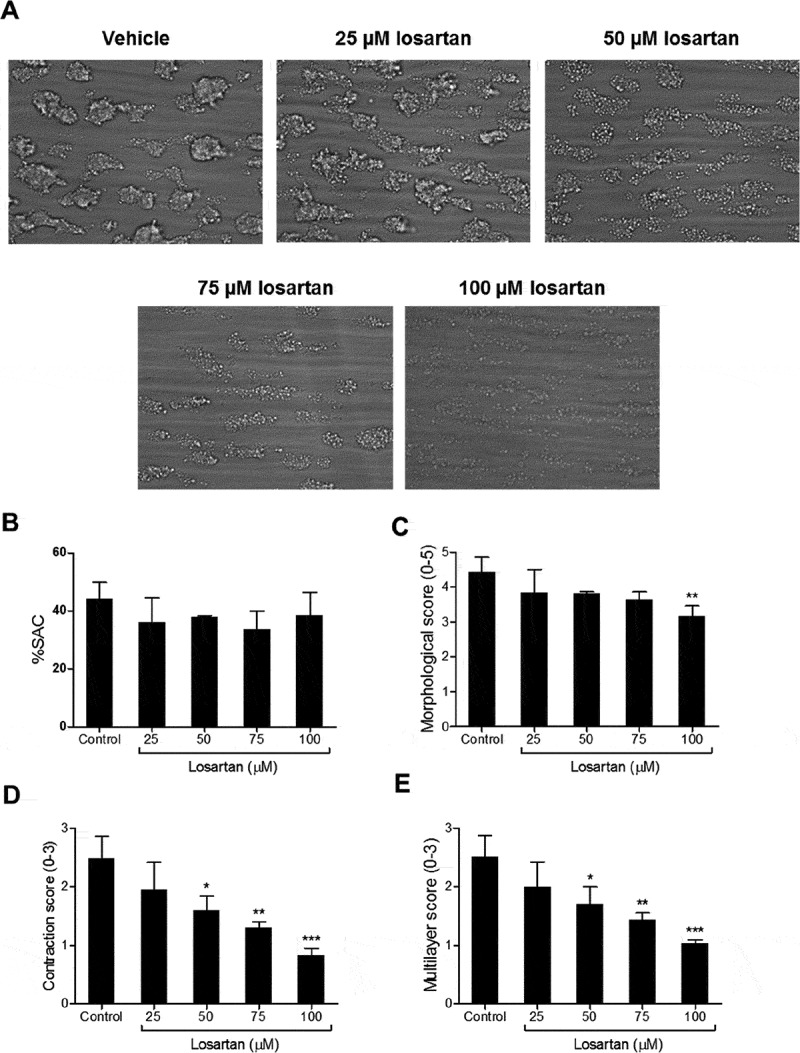
Losartan inhibits the formation of contracted, multilayered platelet thrombi under flow. Whole blood treated with vehicle solution or losartan was perfused over collagen surface for 6 min at wall shear rate of 1000 s^−1^. Phase contrast images were captured and shown as representative images a. Platelet surface area coverage (% SAC), thrombus morphological score (range 0–5), thrombus contraction score (0–3) and platelet multilayer score (0–3) were determined b-e. Data are presented as mean ± SEM (n = 4). *P < .05, **P < .01, ***P < .001.

**Figure 7.**
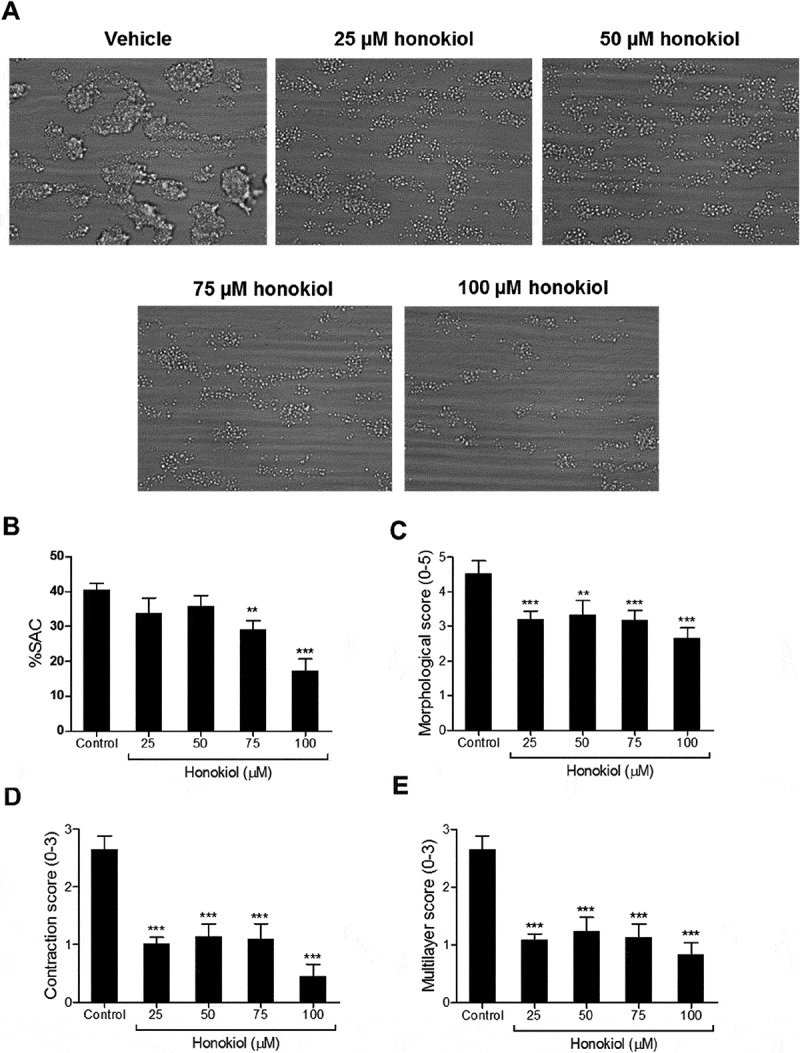
Honokiol inhibits both platelet adhesion and formation of multilayered platelet thrombi under flow. Whole blood treated with vehicle solution or honokiol was perfused over collagen type I for 6 minutes at wall shear rate of 1000 s^−1^. Phase contrast images were captured and shown as representative images a. Platelet surface area coverage (% SAC), thrombus morphological score (range 0–5), thrombus contraction score (0–3) and platelet multilayer score (0–3) were determined b-e. Data are presented as mean ± SEM (n = 4). *P < .05, **P < .01, ***P < .001.

Whole blood perfusion experiments were performed on collagen-coated microspots to assess the effects of platelet pre-treatment with losartan or honokiol (25–100 µM). Thrombi were analysed for platelet deposition (% surface area coverage) and by visual inspection using a pre-defined score system (morphological, contraction and multilayer score) [33]. Losartan treatment did not affect platelet deposition (Figure 6a,b) but decreased thrombus contraction and multilayer scores (Figure 6d,e) as an indication of lower thrombus integrity and the prpesence of loose platelet aggregates. In contrast, honokiol pre-treatment resulted in a dose-dependent decrease in platelet adhesion (lower surface area coverage of platelets) to collagen and significantly decreases the contraction and multilayer scores (Figure 7), pointing to a suppressed formation of tightly packed thrombi. These results show that both losartan and honokiol affect formation of thrombi in whole blood but in different ways. These concentrations of the two inhibitors have little effect however on the response to GPVI activation of platelets in platelet rich plasma due to protein binding (see above).

## Discussion

This work provides new information on the platelet inhibition mechanisms of losartan and honokiol. We confirmed that losartan and honokiol inhibit platelet aggregation by collagen. However, while the IC50 for losartan was similar to that previously reported [21], the IC50 for honokiol is almost one order of magnitude higher than in the original report [26]. The explanation for this difference is unclear. Interestingly, both losartan and honokiol inhibited platelet activation by the (hem)ITAM receptors rhodocytin and FcγRIIA at similar concentrations to those for inhibition by collagen. This effect was mediated by inhibition of tyrosine phosphorylation, including Syk which plays a proximal role in signalling by all three receptors. Inhibition of the response to GPVI was not due to a direct competition with collagen since losartan did not block binding of recombinant GPVI to collagen [15] and honokiol inhibited this binding at a concentration that is three orders of magnitude greater than required for inhibition of aggregation [25].

Although both losartan and honokiol inhibited platelet aggregation and Syk phosphorylation, they were unable to prevent clustering of GPVI or spreading on collagen at concentrations up to 100 µM. This may be related to the relatively high concentration of collagen on the surface thus overcoming the inhibitory effect of the two reagents. Alternatively, neither agent may be able to inhibit clustering of GPVI on a surface and therefore inhibit activation, in contrast to previous results in suspension using an antibody that recognises the dimeric form of GPVI (9E18) conjugated with Duolink PLA probes [15]. Losartan had no effect on constitutive signalling by GPVI in transfected DT40 cells, which may be a functional readout for clustering of the immunoglobulin receptor.

Losartan and honokiol are highly protein bound in plasma [18,32,34] necessitating use of higher concentrations in the presence of plasma to achieve the same level of inhibition as seen in washed platelets. Unexpectedly however, both inhibitors suppressed collagen-dependent platelet aggregation in whole blood under flow conditions at concentrations that hardly affect platelet aggregation in plasma as measured by light transmission aggregometry. Furthermore, the mode of inhibition under flow was distinct for the two inhibitors: while both suppressed the formation of tight platelet thrombi, honokiol also interfered with platelet adhesion. One explanation for the inhibition observed in whole blood could be based on their lipophilic characteristic causing disruption of lipid-to-lipid or lipid-protein interactions [35,36] or blocking protein-protein interactions. In addition, honokiol has been reported to disrupt the integrity of the inner membrane of mitochondria [37]. These data could explain the pleiotropic impacts on various cells in different pathologies [38].

In this study, we compared two small molecules described as specific inhibitors for GPVI on different aspects of platelets activation and function. Although both compounds were able to inhibit platelet aggregation induced by collagen, we could not confirm the original potency of honokiol on this response and we found that neither agent inhibited spreading of platelets on collagen. The likely explanation is that both comounds are unable to inhibit clustering of GPVI of platelets on a surface. Both inhibitors also blocked rhodocytin and FcγRIIA-induced CLEC-2 activation demonstrating that they are not specific to GPVI at the concentrations used. This is illustrated by the inhibition of platelet aggregation on whole blood under flow conditions at concentrations that have a minimal effect on platelet activation by collagen in plasma. The multiplicity of effects of the two inhibitors should be considered in the context of their use as anti-platelet agents.
