# Supplementary material for: Comparison of the GPVI inhibitors losartan and honokiol
Source: Platelets. 2019 Mar 8;31(2):187–97. doi: 10.1080/09537104.2019.1585526 (PMC7034533; doi:10.1080/09537104.2019.1585526)
Supplement: Supplemental Material [file IPLT_A_1585526_SM1723.docx]

**Supplemental Figures**

**
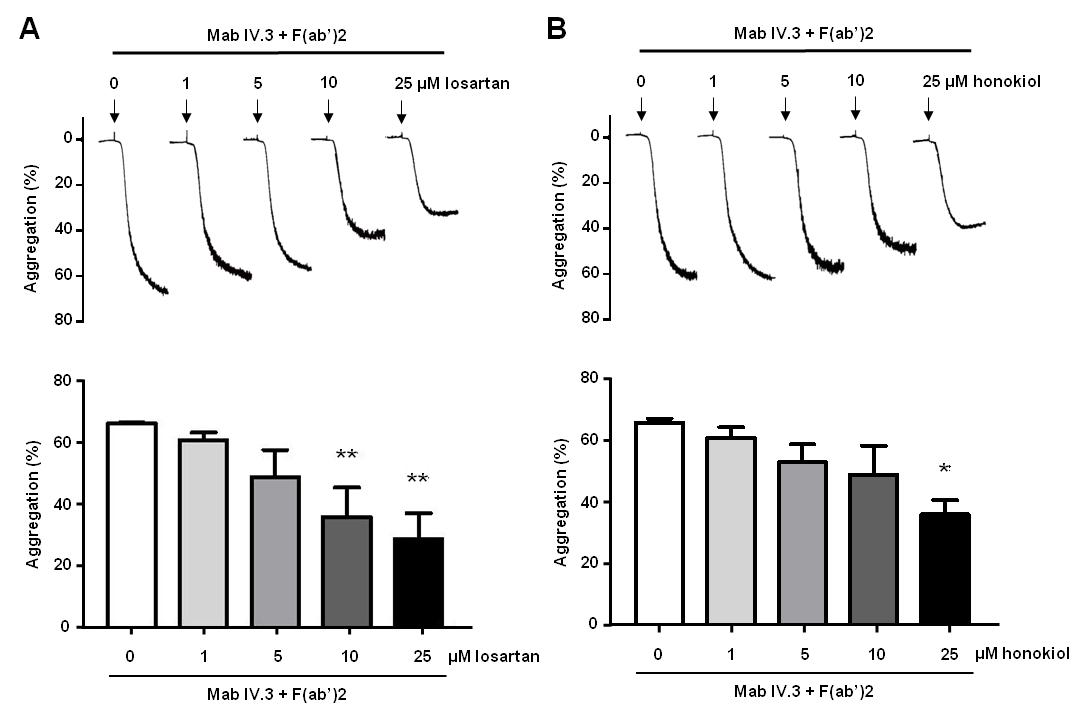
**

**Supplemental Figure 1. Losartan and honokiol dose-dependently reduce aggregation of washed platelets induced by FcγRIIA activation.**

Washed platelets were pretreated with different concentrations of losartan (A) or honokiol(B) for 3 min before addition of mab IV.3 (1 µg/ml) for 60 sec followed by stimulation with F(ab’)_2_ (30 µg/ml). Bar graphs represent a total of 3 independent experiments and results are shown as mean ± SD. *P < .05, **P < .01.

**Supplemental Figure 2. Losartan and honokiol dose-dependently reduce platelet aggregation in plasma, induced by collagen.**

Platelet rich plasma was pretreated with different concentrations of losartan (A) or honokiol (B) for 3 minutes before stimulation with collagen 1µg/ml. The bar graphs represent a total of 5 independent experiments. The results are shown as mean ± SD. **P < .01, ***P < .001. Aggregation traces are representative for the effect of 25µM of losartan (Ci) or honokiol (Cii) on collagen-induced platelet stimulation. The slight delay in aggregation onset observed in the presence of honokiol was not seen in all experiments.


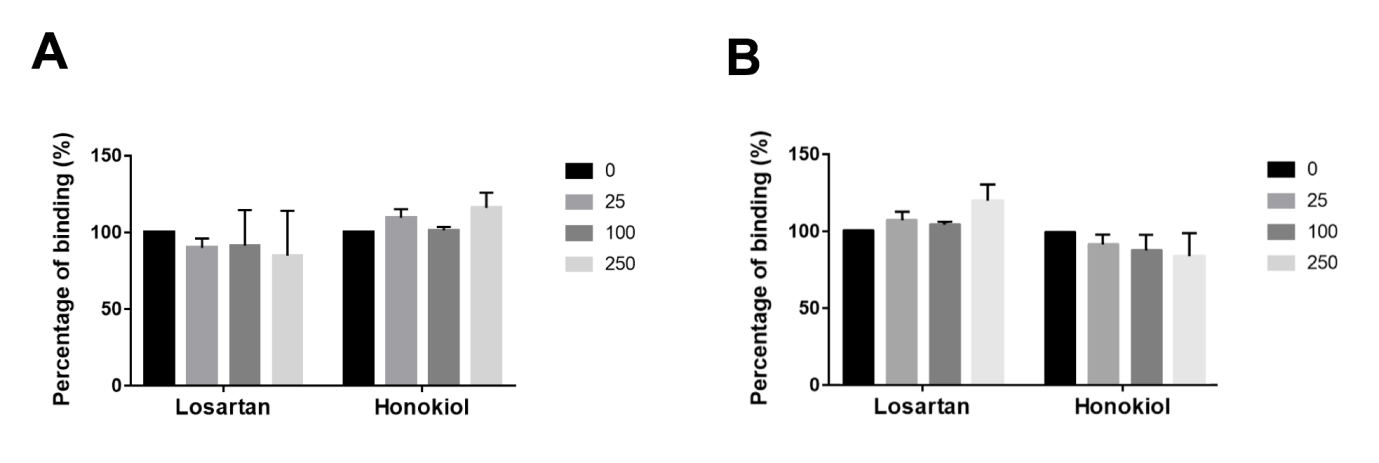


**Supplemental Figure 3. Losartan and honokiol do not inhibit binding of monomeric GPVI to collagen.**

Recombinant GPVI monomeric protein (A) or GPVI dimeric protein (B) was incubated with different concentrations of losartan or honokiol (in µM) before addition onto a collagen coated-surface (10µM). The bar graph represents the binding of the recombinant GPVI to collagen from an ELISA inhibition assay (n = 4). Data are shown as mean ± SD.
